# Seroprevalence and Epidemiological Insights into Severe Fever with Thrombocytopenia Syndrome on Jeju Island, Republic of Korea

**DOI:** 10.3390/v17040466

**Published:** 2025-03-25

**Authors:** Kye-Hyung Kim, Ahreum Kim, Maengseok Noh, Changhoon Kim, Hyunjin Son, Mee Kyung Ko, Jongyoun Yi

**Affiliations:** 1 Department of Internal Medicine, Pusan National University School of Medicine, Busan 49241, Republic of Korea; khkimmd@pusan.ac.kr; 2 Biomedical Research Institute, Pusan National University Hospital, Busan 49241, Republic of Korea; ar_kim_@naver.com (A.K.); kchprev@pusan.ac.kr (C.K.); qeqazwsx@hanmail.net (M.K.K.); 3 Major of Big Data Convergence, Pukyong National University, Busan 48513, Republic of Korea; msnoh@pknu.ac.kr; 4 Department of Preventive Medicine and Occupational and Environmental Medicine, Pusan National University School of Medicine, Busan 49241, Republic of Korea; 5 Department of Preventive Medicine, Dong-A University School of Medicine, Busan 49201, Republic of Korea; hjson@dau.ac.kr; 6 Department of Laboratory Medicine, Pusan National University School of Medicine, Busan 49241, Republic of Korea

**Keywords:** severe fever with thrombocytopenia syndrome, seroprevalence, Jeju Island, tick-borne disease, epidemiology, public health, regional analysis

## Abstract

Severe fever with thrombocytopenia syndrome (SFTS) is an emerging tick-borne disease caused by the SFTS virus, posing significant public health challenges in East Asia. This study aimed to evaluate the seroprevalence of SFTS on Jeju Island, Korea, and to identify the demographic and geographic factors influencing exposure to the virus. A total of 1001 serum samples collected from healthy individuals between 2009 and 2016 were analyzed using a double-antigen enzyme-linked immunosorbent assay. The overall seroprevalence was 1.7%, with slightly higher rates observed in females (2.06%) than in males (1.29%); however, this difference was not statistically significant. Seroprevalence increased with age, peaking at 2.50% in individuals over aged 60 and over. Regional analysis revealed elevated seroprevalence in the eastern coastal areas (4.41%), which was attributed to population density and environmental factors favoring human–tick interactions. These findings suggest that population distribution and land use patterns, rather than altitude alone, significantly affect the exposure of SFTS on Jeju Island. Targeted tick control strategies and public health interventions that focus on high-risk regions and demographics could mitigate SFTS transmission. This study provides valuable insights into the epidemiological characteristics of SFTS and emphasizes the importance of tailored preventive measures in endemic regions.

## 1. Introduction

### 1.1. Background

Severe fever with thrombocytopenia syndrome (SFTS) is an emerging tick-borne viral disease caused by the SFTS virus (SFTSV), also called Dabie bandavirus, a member of the Bunyavirales order [1]. It has been responsible for numerous cases in East Asia, particularly in countries such as Korea, China, and Japan [1,2,3]. SFTS was first identified in China in 2009 and has since become a major public health concern because of its high mortality rate and severe clinical manifestations, including fever, thrombocytopenia, leukopenia, and multi-organ dysfunction [4]. This disease primarily affects individuals engaged in outdoor occupations, especially those frequently exposed to tick-infested environments [5]. In South Korea, SFTS has been observed more frequently in rural and agricultural areas where tick populations, particularly *Haemaphysalis longicornis*, are abundant.

Jeju Island, an area known for its rural landscapes and active agricultural sector, has also reported sporadic cases of SFTS over the past decade [6]. Notably, Jeju Island had the highest SFTS incidence, 12.7 per 100,000 by percentage of the population [7]. Although absolute case numbers remain lower than in mainland regions, Jeju’s environmental and occupational risk factors may contribute to localized transmission dynamics. Understanding the regional seroprevalence of SFTS in Jeju provides valuable epidemiological insights for targeted public health interventions [6]. SFTSV has been detected in various tick species, particularly *Haemaphysalis longicornis*, the primary vector in Republic of Korea. A study conducted on Jeju Island reported a minimum infection rate (MIR) of SFTSV in ticks at 0.37%, which is lower than the 1.97% MIR observed in other regions. This indicates a relatively lower prevalence of SFTSV in ticks on Jeju Island compared to other areas. Given the island’s unique environmental conditions and its high potential for tick exposure, further surveillance is necessary to assess the risk of tick-to-human transmission in this region. Therefore, Jeju Island offers a valuable opportunity to study the epidemiological patterns of SFTS in endemic areas. Monitoring the seroprevalence of SFTS in this region is crucial for understanding the disease’s transmission dynamics and identifying at-risk populations that may benefit from targeted public health interventions.

### 1.2. Objective

This study aimed to evaluate SFTS seroprevalence rates across various demographic factors, specifically sex, age group, and geographic region—within Jeju Island. Using serum samples collected from healthy individuals undergoing routine health screening between 2013 and 2015, this study aimed to identify the patterns of SFTS exposure across the island’s population. By assessing these data, this study aimed to provide a clearer understanding of the regional transmission dynamics of SFTSV and highlight the areas with a heightened risk of exposure. These insights are intended to aid the development of disease prevention and control strategies and to guide public health authorities to implement measures that mitigate the spread of SFTS within high-risk communities on Jeju Island.

## 2. Materials and Methods

### 2.1. Study Population and Sample Collection

Serum samples were obtained from samples initially collected for health screening purposes at the Jeju National University Hospital Health Examination Center. These samples, which were retained after routine health checkups, were subsequently provided by the the Biobank of Jeju National University Hospital, which facilitated access to a diverse sample population across different demographics. The samples were collected between 2009 and 2016 and processed according to the biobank’s specimen handling guidelines, where serum was separated and aliquoted, then stored at −80 °C until distribution. The samples remained at −80 °C until immediately before ELISA testing, ensuring sample integrity. The inclusion criteria consisted of individuals undergoing health screenings at Jeju National University Hospital, who provided written consent to donate their biospecimens to the Biobank of Jeju National University Hospital. Only samples with documented written consent for both human biospecimen donation and the use of human biospecimens in research were included in the study. Exclusion criteria were applied to ensure data integrity. Samples were excluded if the sample ID was missing or unidentifiable, or if information on sex or age was incomplete or missing. This collection approach allowed for a broad and representative sample of healthy individuals from Jeju Island, encompassing various age groups, sexes, and geographic backgrounds. Duplicate samples were identified and excluded to ensure data integrity. Additionally, the biobank conducted a thorough review to remove any personally identifiable information from the samples before distribution, resulting in a unique dataset comprising 1001 serum samples for the seroprevalence analysis of SFTS. Since the samples were obtained from individuals undergoing routine health checkups, only a limited number of samples were available from individuals in their 20s and younger.

### 2.2. Serological Testing and Double-Antigen Enzyme-Linked Immunosorbent Assay (ELISA) Method

Serological testing for SFTS antibodies was conducted using double-antigen sandwich ELISA, an established technique for detecting antibodies with 100% sensitivity and 99.57% specificity. This assay detects total SFTS-specific antibodies, including immunoglobulin (Ig) G and IgM, which serve as indicators of past or recent exposure to the virus. In this study, a double-antigen ELISA was performed as an in-house assay in the researchers’ laboratory, following the methodology described in previous studies [8]. Briefly, the ELISA involved coating plates with the recombinant SFTS nucleocapsid protein (rNP), followed by the addition of serum samples. After an incubation period, a HRP-conjugated rNP was introduced. Through a chromogenic reaction, producing a measurable color change of SFTS-specific antibodies present in the sample.

All the samples were tested in duplicate to ensure consistency and accuracy, and both positive and negative controls were included in each assay run. The optical density values were read at 450 nm. The results were expressed as a percentage of the positive control (PP), with the cutoff determined as the mean PP plus 3 standard deviations from the negative-control serum [8].

### 2.3. Data Collection and Analysis

Demographic data including age, sex, and geographic location of residence were collected for each participant. Geographic data were further categorized into broader administrative regions (e.g., Jeju City, Seogwipo City, and unspecified areas) and more specific coastal and inland regions based on known tick habitats and potential exposure zones. The data were analyzed to determine the overall seroprevalence rates and examine the differences in SFTS positivity according to sex, age group, and region.

### 2.4. Statistical Analysis

Statistical analyses were conducted using chi-square tests to compare the SFTS seroprevalence across sexes, age groups, and geographic areas. For the age-specific analysis, the samples were grouped into 10-year intervals to assess trends across different life stages. Additionally, the Cochran–Armitage test for trend was performed to evaluate potential trends in antibody prevalence across age groups, with the significance level set at a *p*-value of <0.05. General characteristics were evaluated using SAS 9.4 (SAS Institute Inc., Cary, NC, USA), with statistical significance set at a *p*-value of <0.05. Logistic regression was performed to identify potential risk factors for seropositivity, controlling for demographic variables, to assess whether certain characteristics, such as age or geographic location, significantly influenced SFTS exposure risk.

The results of these analyses provide insights into the seroprevalence distribution across Jeju Island and offer valuable data for guiding future public health interventions.

### 2.5. Ethical Statement

This study was approved by the Institutional Review Board (IRB) of Pusan National University Hospital (PNUH IRB No. E-2015087). Written informed consent was obtained from the participants for both the human biospecimen donation and the use of human biospecimens in research.

## 3. Results

### 3.1. Overall Seroprevalence

Of the 1001 serum samples tested, 17 tested positive for SFTS antibodies, resulting in an overall seroprevalence of 1.7%. This finding indicates a low but notable exposure of the population on Jeju Island to SFTSV. Although the overall seroprevalence remains below 2%, the detection of positive cases in a healthy population emphasizes the silent circulation of the virus in the region. This prevalence likely reflects a combination of environmental risk factors, occupational exposure, and the ecological presence of *Haemaphysalis longicornis* ticks, which are known vectors of the virus.

### 3.2. Sex- and Age-Specific Seroprevalence

The sex-specific analysis revealed a slightly higher seroprevalence among females (2.06%) than among males (1.29%) (Table 1). Although this difference was not statistically significant (*p* = 0.4638), it may indicate sex-specific variations in exposure risk, possibly owing to differences in occupational or recreational activities.

The age-specific analysis demonstrated that the highest seroprevalence was observed in participants aged ≥60 years (2.50%), followed by those in their 50s (1.97%) and 40s (1.70%). However, the Cochran–Armitage test for trend indicated that this observed trend with advancing age was not statistically significant (*p* = 0.9095).

### 3.3. Regional Seroprevalence

Among the total 1001 participants, 983 individuals had a confirmed residence in Jeju, while 6 individuals with unconfirmed residency and 12 classified as residing outside Jeju were excluded from the regional seroprevalence comparison. The geographic analysis revealed comparable seroprevalence rates between Jeju City (1.75%) and Seogwipo City (1.64%), with no significant differences (*p* = 0.8489) (Table 2). The absence of positive cases in other or unspecified regions further underscores the potential clustering of SFTS exposure in the urban or suburban areas of Jeju Island.

A more detailed geographic analysis of coastal and inland regions highlighted the regional differences. The eastern coastal area had the highest seroprevalence (4.41%), followed by general Jeju coastal areas (1.73%) and Seogwipo coastal areas (1.60%). In contrast, no positive cases were identified in western coastal or central inland regions. Although these differences were not statistically significant (*p* = 0.5418), the elevated seroprevalence in the eastern coastal area suggests potential environmental or occupational factors that warrant further investigation.

### 3.4. Seroprevalence by Detailed Administrative Divisions (Eup/Myeon/Dong)

**Figure 1:**
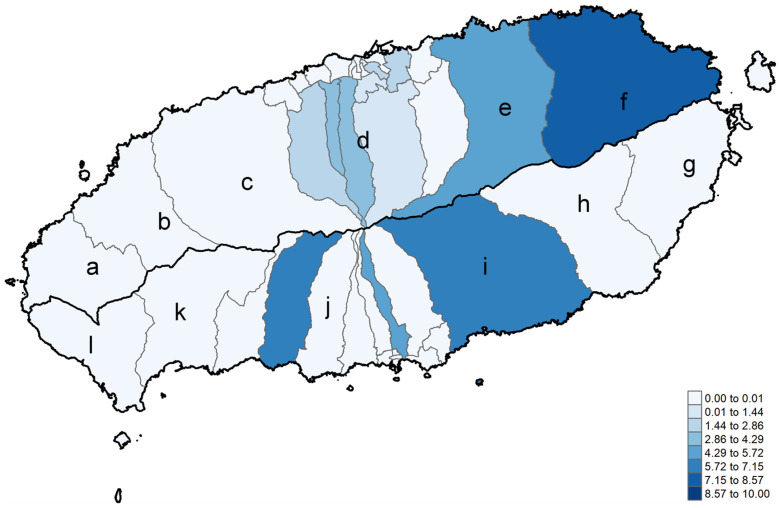
Distinct areas within Jeju Island (Figure). This figure represents the SFTS seroprevalence rates in different regions of Jeju Island, Republic of Korea, based on 983 participants with confirmed residence in Jeju. The seroprevalence rates were measured using a double-antigen ELISA and are depicted across distinct areas of the island. The regions shown in the figure include (**a**) Hankyung-myeon (n = 4), (**b**) Hallim-eup (n = 12), (**c**) Aewol-eup (n = 47), (**d**) Jeju-City, dong area, excluding myeon and eup (n = 701), (**e**) Jocheon-eup (n = 22), (**f**) Gujwa-eup (n = 13), (**g**) Seongsan-eup (n = 8), (**h**) Pyoseon-myeon (n = 7), (**i**) Namwon-eup (n=15), (**j**) Seogwipo-City, dong area, excluding myeon and eup (n = 128), (**k**) Andeok-myeon (n = 9), and (**l**) Daejeong-eup (n = 16). The numbers in the lower right corner represent seroprevalence rates for each respective region.

The results indicated varying seroprevalences across these divisions (Figure 1). Notably, Namwon-eup in Seogwipo City exhibited a relatively high seroprevalence of 6.67%, with 1 out of 15 participants testing positive. Similarly, Gujwa-eup in Jeju City showed a seroprevalence of 7.69%, with 1 positive case among 13 participants. Other areas, such as Donghong-dong in Seogwipo City and Jocheon-eup in Jeju City, also reported moderately elevated seroprevalence of 4.55%. In contrast, many regions, including the majority of central inland areas and several coastal regions, had no positive cases, suggesting the potential clustering of SFTS exposure in specific localities.

To assess the potential differences in SFTS exposure between urban and rural populations, the sample was divided into Dong (urban) and Eup/Myeon (rural) areas. The results, summarized in Table 3, show that in urban areas (Dong), 14 out of 829 participants tested positive, resulting in a seroprevalence of 1.69%. In rural areas (Eup/Myeon), 3 out of 154 participants tested positive, with a slightly higher seroprevalence of 1.95%. However, the difference was not statistically significant (*p* = 0.7395). These findings suggest that, on Jeju Island, the risk of SFTS exposure is not significantly influenced by the urban or rural status of the area, although environmental factors unique to specific locales might play a role.

## 4. Discussion

This study provides a comprehensive evaluation of the seroprevalence of SFTS on Jeju Island, offering valuable insights into the demographic and geographic factors that affect exposure to SFTSV. These findings emphasize the need for targeted interventions in high-risk populations and regions.

The overall seroprevalence rate of 1.7% observed in this study aligns with other studies conducted in rural regions of Korea, where seroprevalence typically ranges from 1% to 3%, depending on the population studied [9]. For example, a nationwide survey using data from the Korea National Health and Nutrition Examination Survey found comparable seroprevalence in rural populations [10]. This suggests that Jeju Island shares similar risk profiles with other agricultural and rural areas in Korea, where outdoor exposure to tick habitats is prevalent. Globally, the seroprevalence in this study is consistent with those reported in endemic regions of China but higher than those observed in Japan, where public health measures and environmental factors may differ [8,11,12,13].

Although not statistically significant, the slightly higher seroprevalence in females (2.06% vs. 1.29% in males) could be linked to sex-specific differences in outdoor activities, such as gardening and farming, which may increase exposure to tick habitats [9,14,15,16]. Further studies investigating activity patterns could provide clarification. Although our study did not collect specific data on participants’ occupations, previous research has suggested that individuals engaged in farming and outdoor activities may have an increased risk of SFTS exposure due to frequent contact with tick-infested environments [17]. Given that Jeju Island has a significant agricultural sector, the potential for occupational exposure remains an important consideration for future studies. Our findings emphasize the need for targeted public health interventions, particularly in populations with a higher likelihood of frequent outdoor exposure, rather than assuming occupational risk based solely on demographic or regional factors.

The age-based analysis indicated an increasing trend in seroprevalence with advancing age, particularly in the 50s and those aged 60 and older groups, although this trend was not statistically significant. The highest seroprevalence of 2.50% was observed in individuals ≥ 60 and older, which may reflect cumulative exposure due to the prolonged time spent in tick-infested areas. However, the higher seroprevalence observed in older age groups may not be solely attributable to cumulative exposure over time [9]. SFTSV-specific IgG antibodies can reportedly diminish within a few years after infection, with one study reporting that antibodies disappeared approximately 44 months after infection [18]. This finding indicates that the higher seroprevalence among older adults may also reflect ongoing or repeated exposure rather than solely the lifetime accumulation of risk. In addition, older adults in rural areas are more likely to engage in outdoor activities or farming, which further increases the risk of repeated tick exposure.

The regional analysis revealed the highest seroprevalence in the eastern coastal areas, where 4.41% of participants tested positive, although this difference was not statistically significant. This elevated seroprevalence may reflect the unique geographic and demographic characteristics of Jeju Island. Coastal areas below 200 m account for 55.3% of the island’s total area and host the majority of its population, increasing opportunities for human–tick interactions due to vegetation and livestock farms [19]. In contrast, mid-altitude regions (200–500 m), which are less populated and primarily consist of pasturelands, showed no positive cases. These findings differ from those of nationwide studies conducted in Korea, which reported a higher incidence of SFTS in hilly regions. This suggests that population distribution and land use patterns, rather than altitude alone, play a critical role in determining SFTS exposure on Jeju Island. Localized tick control efforts targeting coastal regions can help mitigate the risk of SFTS.

Despite the slightly higher seroprevalence in rural areas (Eup/Myeon) than in urban areas (Dong), the difference was not statistically significant. This trend may reflect population density patterns, as even urban residents on Jeju Island frequently engage in outdoor activities, thereby exposing them to tick habitats. As such, public health interventions should not solely target rural populations but also consider urban communities engaged in outdoor leisure or agricultural activities.

This study has several limitations. First, its cross-sectional nature limits the ability to determine causality or seasonal variations in seroprevalence. Longitudinal studies can provide insights into the persistence of SFTS antibodies and seasonal exposure trends. Additionally, this study did not collect detailed data on the participants’ specific occupations, outdoor activity levels, or tick exposure history. Such information could provide valuable insights into the behavioral and environmental risk factors that influence seroprevalence. Research into tick ecology and environmental factors in areas with high tick prevalence could lead to more effective localized control measures.

## 5. Conclusions

This study provides valuable insights into the seroprevalence of SFTS on Jeju Island and identifies specific demographic and geographic trends that highlight potential risk factors for SFTSV exposure. Although the overall seroprevalence was low, our findings suggest that targeted public health interventions, especially in high-risk coastal and rural areas, may play a vital role in reducing the incidence of SFTS. Raising awareness and promoting protective behaviors among outdoor workers and older adults on Jeju Island may further help mitigate the risks associated with this emerging tick-borne disease.

## Figures and Tables

**Table 1:** Sex- and Age-Specific Seroprevalence of SFTS.

Demographic Group	Total Samples (N)	Positive Samples (N)	Seropositivity Rate (%)	*p*-Value
Sex				
Male	466	6	1.29	0.4638
Female	535	11	2.06	
Age Group (years)				0.2358 *
10–19	1	0	0.00	
20–29	30	0	0.00	
30–39	185	2	1.08	
40–49	411	7	1.70	
50–59	254	5	1.97	
≥60	120	3	2.50	

* *p*-value using Cochran–Armitage test for trend.

**Table 2:** Regional Seroprevalence of SFTSV among 983 Participants with Confirmed Residence in Jeju.

Region	Total Samples (N)	Positive Samples (N)	Seropositivity Rate (%)	*p*-Value
Broad Region				
Jeju City	800	14	1.75	0.8489
Seogwipo City	183	3	1.64	
Detailed Region				
Eastern Coastal Area	68	3	4.41	0.5418
Western Coastal Area	77	0	0.00	
Central mid-mountain area	21	0	0.00	
Jeju Coastal Area	692	12	1.73	
Seogwipo Coastal Area	125	2	1.60	

**Table 3:** Comparison of SFTS Seroprevalence Between Urban (Dong) and Rural (Eup/Myeon) Areas in Jeju Island Among 983 Participants with Confirmed Residence.

Region	Total Samples (N)	Positive Samples (N)	Seropositivity Rate (%)	*p*-Value
Urban (Dong)	829	14	1.69	0.7395
Rural (Eup/Myeon)	154	3	1.95	

## Data Availability

The datasets generated and/or analyzed in the current study are available from the corresponding author upon reasonable request.

## Abbreviations

The following abbreviations are used in this manuscript:

ELISA	Enzyme-linked immunosorbent assay
SFTS	Severe Fever with Thrombocytopenia Syndrome
SFTSV	SFTS virus
